# Application of multidirectional stitching technology in a laparoscopic suturing instructional program: a randomized controlled trial

**DOI:** 10.1186/s12909-020-02146-w

**Published:** 2020-08-04

**Authors:** Yu Zhao, Qiong Chen, Jia-Ning Hu, Qi Shen, Lu Xia, Lin-Zhi Yan, Yi Wang, Xiu-Jie Zhu, Wen-Ju Li, Yue Hu, Qiong Zhang

**Affiliations:** 1 Department of Obstetrics and Gynaecology, The Second Affiliated Hospital and Yuying Children’s Hospital of Wenzhou Medical University, Wenzhou, Zhejiang, 325000 China; 2 The Second School of Medicine, Wenzhou Medical University, Wenzhou, Zhejiang, 325000 China

**Keywords:** Multidirectional stitching technology, Laparoscopic suturing, Instructional program

## Abstract

**Background:**

Surgeon suturing technology plays a pivotal role in patient recovery after laparoscopic surgery. Intracorporal suturing and knot tying in minimally invasive surgery are particularly challenging and represent a key skill for advanced procedures. In this study, we compared the application of multidirectional stitching technology with application of the traditional method in a laparoscopic suturing instructional program.

**Methods:**

We selected forty residents within two years of graduation to assess the specialized teaching of laparoscopic suturing with laparoscopic simulators. The forty students were randomly divided into two groups, a control group and an experimental group, with twenty students in each group. The control group was scheduled to learn the traditional suture method, and the experimental group applied multidirectional stitching technology. The grades for suturing time, thread length, accuracy of needle entry, stability of the knot, tissue integrity, and tightness of the tissue before and after the training program were calculated.

**Results:**

There was no significant difference between the two groups before the learning intervention. After the program, both groups significantly improved in each subject. There were significant differences between the control group and the experimental group in suture time (*P* = 0.001), accuracy of needle entry and exit (*P* = 0.035), and whether the suture tissue had cracks (*P* = 0.030). However, the two groups showed non-significant differences in thread length (*P* = 0.093), stablity of the knot (*P* = 0.241), or tightness of the tissue (*P* = 0.367).

**Conclusions:**

Multidirectional stitching technology improves the efficiency and effectiveness of traditional laparoscopic suture instructional programs. It might be a practicable, novel training method for acquiring proficiency in manual laparoscopic skills in a training setting.

## Text

Application of multidirectional stitching technology in a laparoscopic suturing instructional program: a randomized controlled trial.

## Background

As the concept of minimal invasiveness has persisted, and the instrumentation for minimally invasive surgery has been developed and adopted widely, laparoscopy has been performed in clinical work pervasively and gained increasing popularity among surgeons [1]. Intracorporal suturing and knot tying in minimally invasive surgery is particularly challenging and represents a key skill for advanced procedures [2]. It determines the speed of the operation and the amount of bleeding during the operation, and directly affects the healing of surgical wounds [3]. Therefore, how to improve laparoscopic suturing technique is of great significance to enhance the efficiency and to speed up the recovery of laparoscopic minimally invasive surgery.

Different instructional programs with varying properties and modalities have been developed [4–7]. Advanced simulation-based instructional programs and practice outside the operating room are vital in boosting the efficiency of laparoscopic suturing and shortening the learning curve for junior surgeons [8]. After numerous modifications, we propose a new suturing simulation technology that applies the method of multidirectional needle delivery to promote the application of laparoscopy.

The aim of this study is to compare multidirectional stitching technology in laparoscopic suturing instructional programs and traditional laparoscopic suturing instructional programs. Furthermore, we objectively assess multidirectional stitching technology in laparoscopic suturing instructional programs.

## Methods

**Fig. 1:**
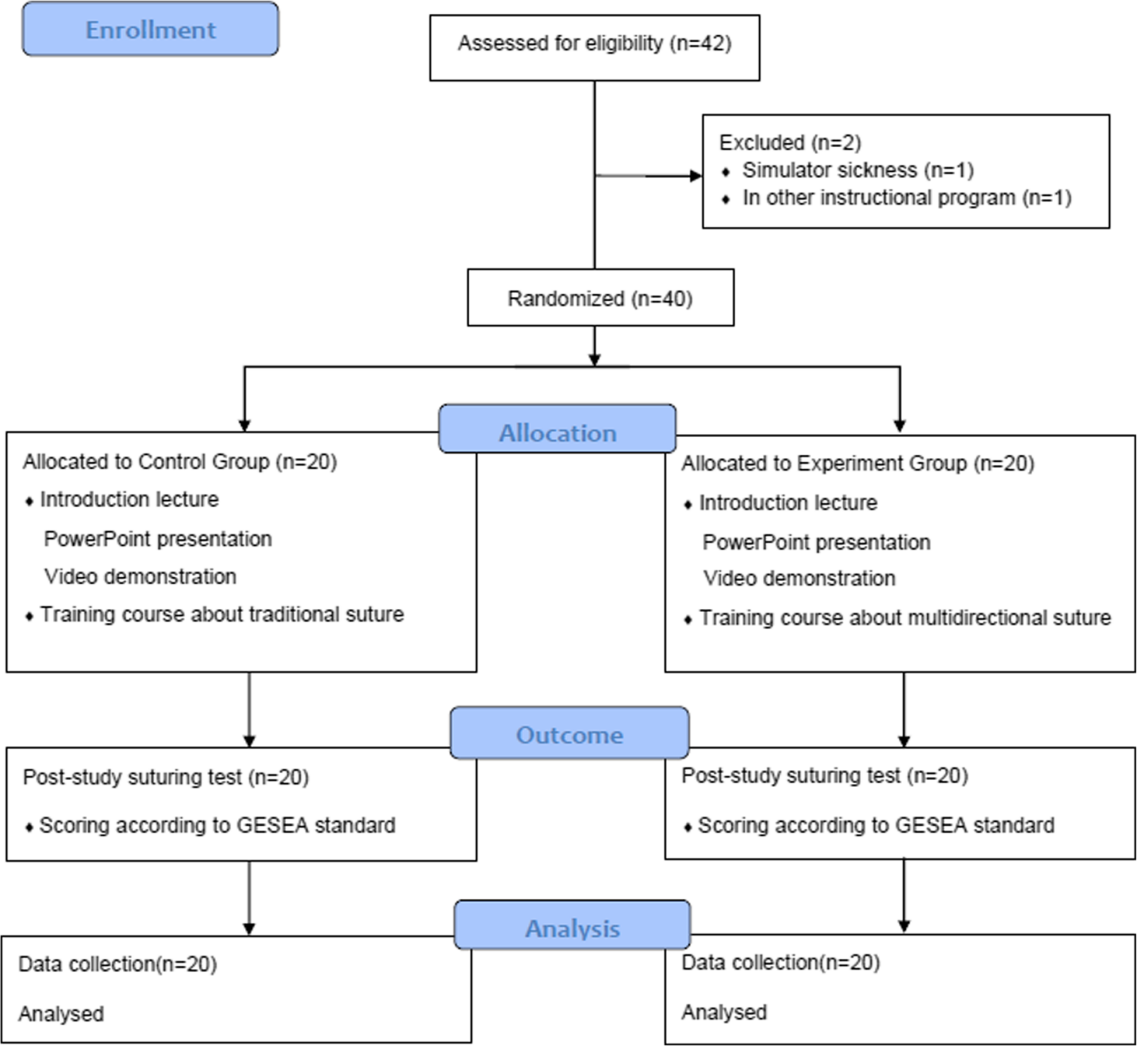
The flow chart of study protocal

The study was designed according to the CONSORT statement (Fig. 1). This was a randomized control trial into which junior residents in The Second Affiliated Hospital of Wenzhou Medical University were enrolled. The inclusion criteria were as follows: novice to laparoscopy, no simulator sickness, and committed to completing the training. Students who had already taken part in other instructional programs at the same time or had no interest in laparoscopy were excluded. Informed consent was obtained from all individual participants.

### Recruitment

All candidates were junior residents informed by flyers, social networks and personal contact. After exclusion, forty volunteers were blindly randomized into a control group and an experimental group by computer-generated simple randomization, with twenty volunteers in each group. One statistical staff in our hospital performed the randomization and allocation process, and ensured allocation concealment. The residents were asked to complete a pre-study questionnaire about their demographic information, such as age, sex and video gaming experience.

### Materials and equipment

**Fig. 2:**
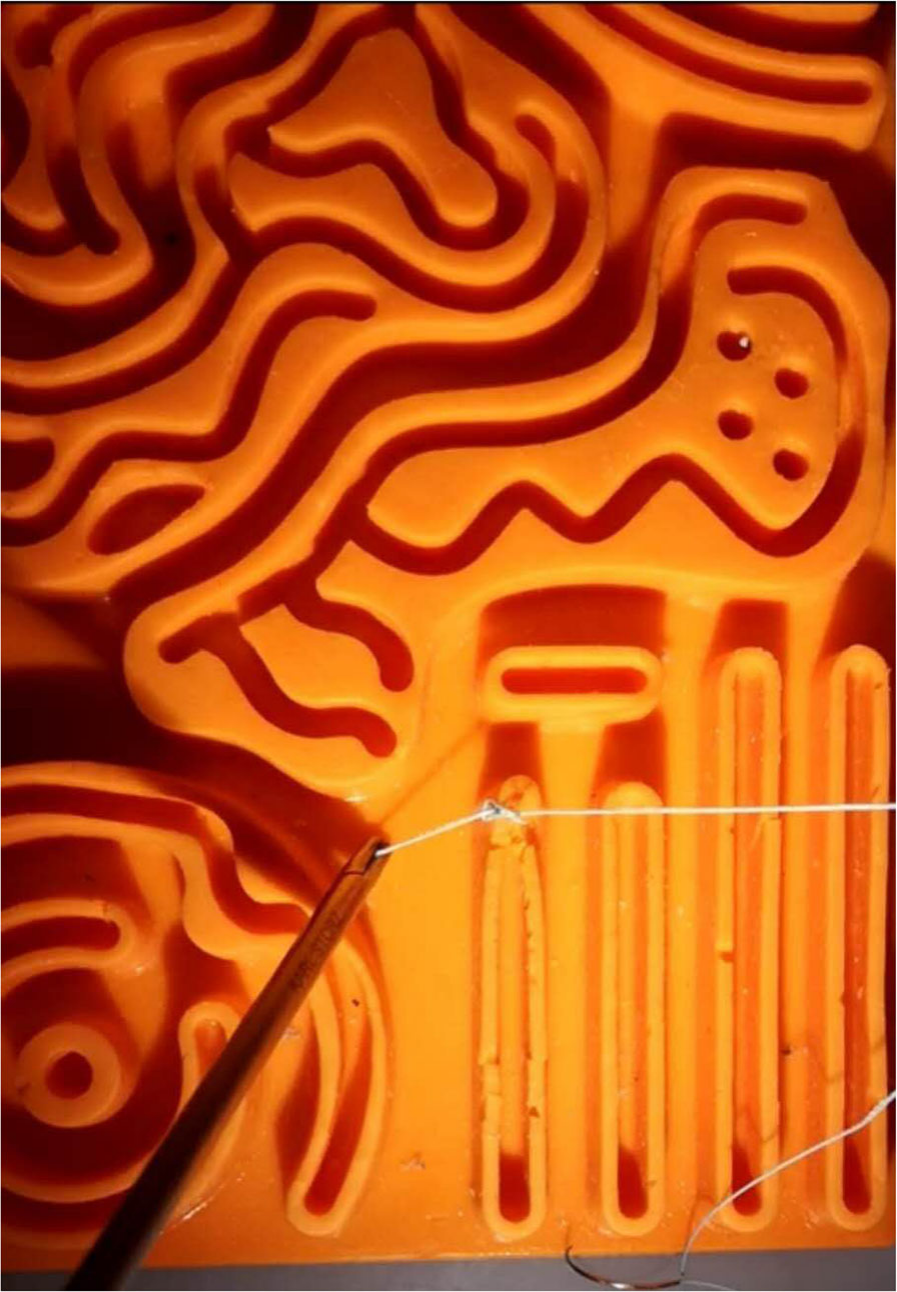
The format of traditional stitch module

**Fig. 3:**
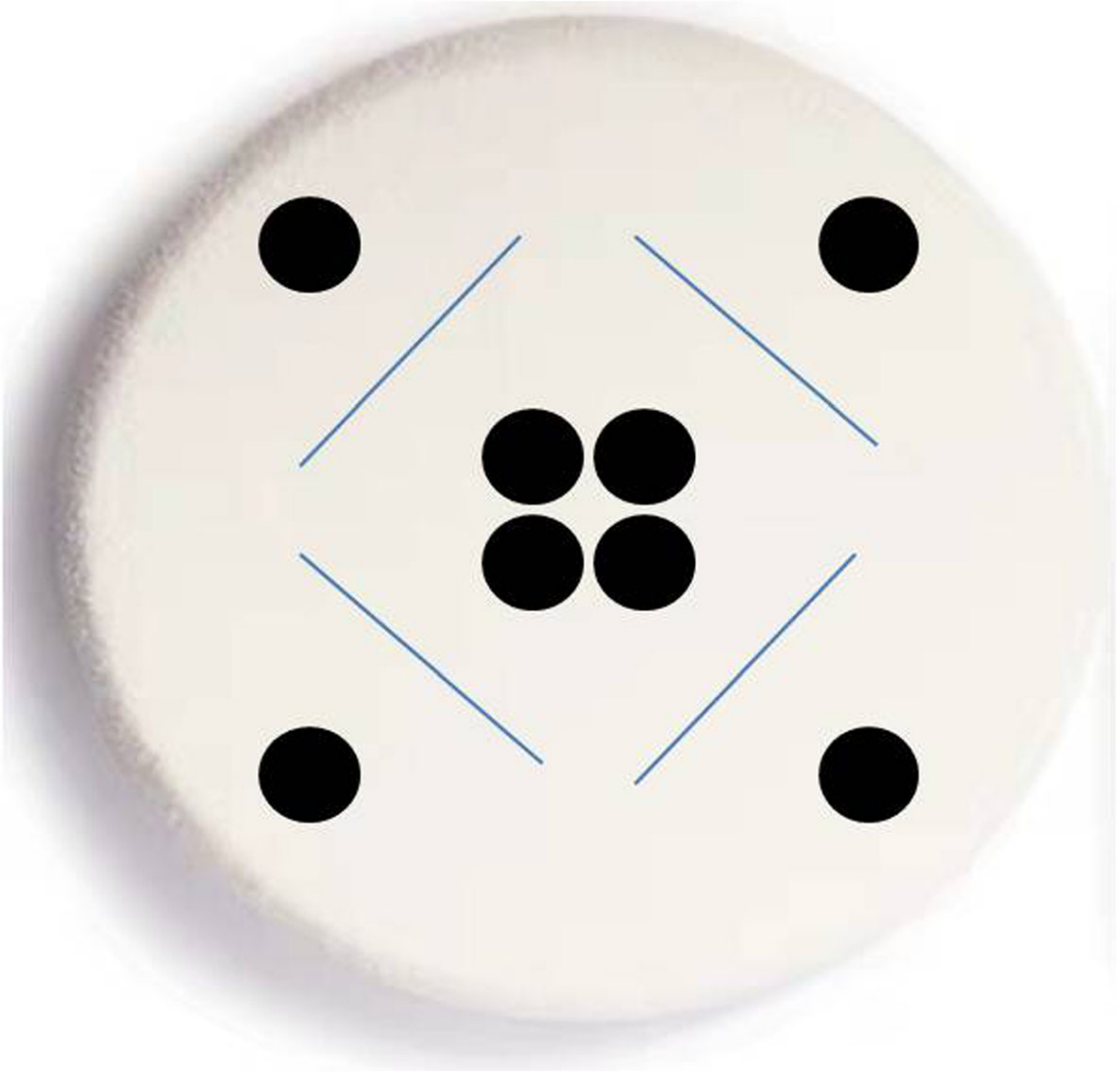
The format of multidirection stitch module

The same laparoscopic trainer box (KARL STORZS Endoscopy Ltd. Germany) was used in the two groups; the trainer box included a camera with video display and nine skin-like access ports for the introduction of laparoscopic instruments. Two lockable laparoscopic needle holders were prepared to complete the suture. A No. 1, 26 mm, 1/2 c round bodied needle with a 30 cm long needle thread (ETHICON.LLC Company) was provided for practice. For the control group, the students were trained with traditional laparoscopic suture instruction on a 3D silicone model pad, as shown in Fig. 2. For the experimental group, we designed a special circular suture training module on a slightly hard sponge material, as shown in Fig. 3. There were four incisions in different directions. We designed the trainer, in which the point where the needle stitched in and out was a small dot with a diameter of 0.5 cm on both sides of the incision. And there was a vertical distance of 0.75 cm from the centre of the dot to the incision. An additional movie file shows the suture in more detail (see Additional File 1).

**Additional file 1.** The process of multidirection stitching (This additional file shows how the multidirection stitching technology is performed in a video).

### Training sessions

The instructional program consisted of a total of four courses, two courses each week. Every course lasted for forty minutes. Two groups were taught by the same experienced instructor at different time in the same laparoscopic-teaching classroom. The first course was planned as an introduction about laparoscopic surgery, suturing technology, and a video demonstration according to different groups. The following three courses were for hands-on practice under instructor’s guidance.

Traditional laparoscopic suturing refers to the use of laparoscopic lenses, needle holders, bending pliers and other operating instruments to complete the suture and knotting procedures. Generally, the procedure involves a needle holder holding the needle at an angle of 90 degrees using rotation of the wrist and suturing the tissues together with the needle and thread. The suture angle is fixed only at 90 degrees with the needle, and the needle cannot change this at will, which is similar to the method of holding the needle in open surgery.

Laparoscopic multidirectional needle delivery refers to the variety of needle holding directions during the needle holding stage in laparoscopy. It includes a positive needle direction, reverse needle direction, oblique needle direction (left, right), swivel needle direction, and so on. More directional and angular adjustments are applied for holding the needle than are required for open abdominal surgery. For example, for the righthand, the so-called positive needle director indicates holding the needle and putting the needle tip to the left 90 degrees. The reverse needle director indicates holding the needle tip 90 degrees to the right. The angle of the oblique needle is not 90 degrees, less than 90 degrees or more than 90 degrees and is divided into left and right directions. The swivel needle director indicates that the needle holder is incompletely clamping the needle. We call this “loosely clipping” the needle. According to the direction and distance of the suturing target, the direction of the needle is constantly changed during the stitching process, which means the needle is “wandering” in the tissue according to the operators’ will. The location of the needle puncture out from the tissue is not consistent with the direction of the needle puncture into the tissue.

### Outcome parameters

**Fig. 4:**
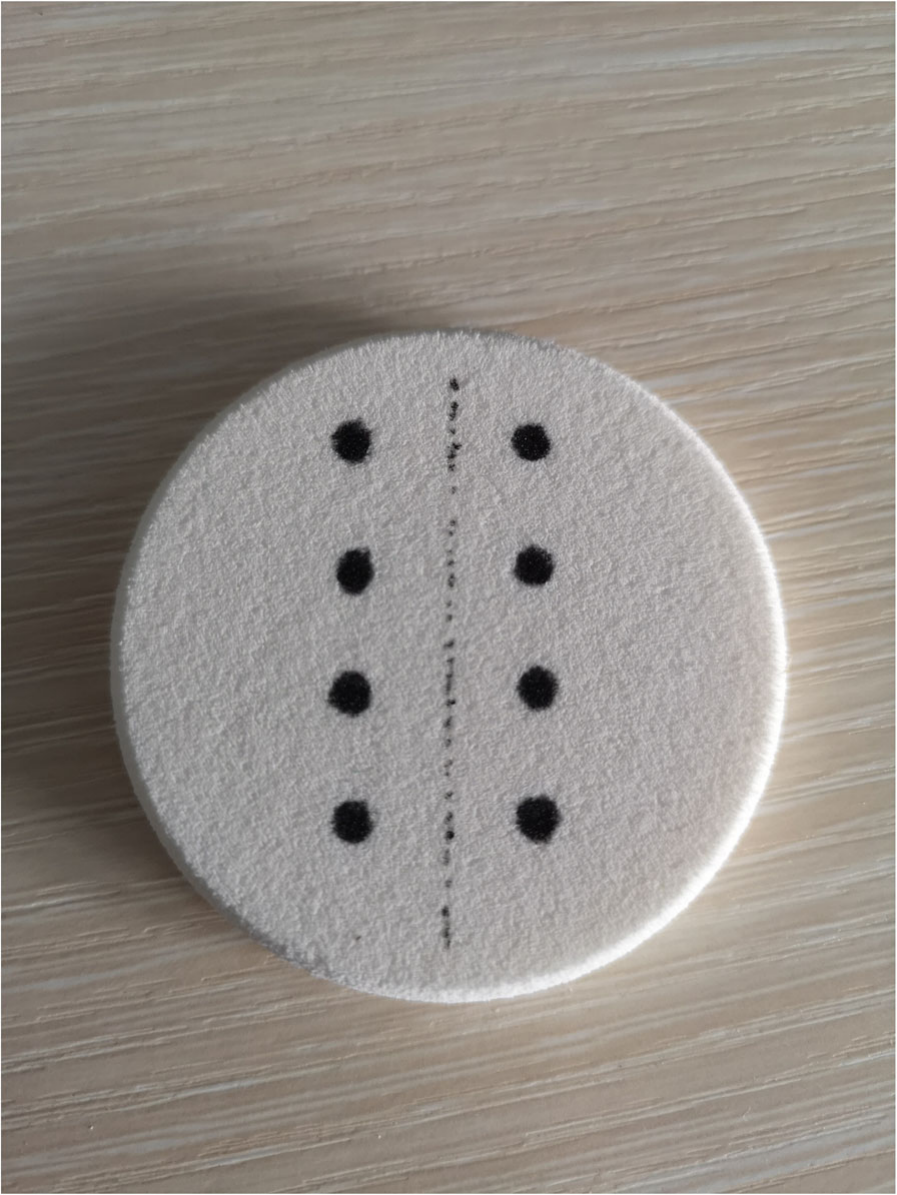
The format of pre-post test module

All candidates were tested with a suture pad with eight marked needle entrance and exit targets before and after training (Fig. 4). The video display on the trainer box recorded their performance. To ensure fairness, two experienced laparoscopic surgeon rated scores by watching the performance video. The following scoring criteria were used: (1) The needle stick should be in and out of the centre of the dot. The operator received 2 points if it was in the centre of the dot, 1 point if it deviated from the centre, and 0 points if it was outside the dot. (2) The knot was required to be a surgical triple knot and should not have been a smooth knot or a loose knot. A normal knot received 2 points. A smooth knot received 1 point, and 0 points were awarded for a loose knot. (3) The suture tissue should not be damaged or ruptured. The trainee received a score of two for integrity, 1 for a few cracks, and 0 for a complete fracture of the tissue. (4) The tightness of the suture tissue indicated that the two sides of the sutured tissue were closely connected. The operator received 2 points if there was no gap between the two sides of the incision, 1 point for a few gaps, and 0 points for a totally loose suture. The total suture time and the length of the suture thread (30 cm minus the remaining suture thread was the final result) was also calculated. This scoring standard is based on the scoring standard of the GESEA [9] (Gynaecological Endoscopic Surgical Education and Assessment) training and evaluation system for gynaecological endoscopic surgery and was slightly modified according to the model we designed (Table 1).

**Table 1:** GESEA-based Scoring Table

Suture time (min)			
Length of the thread (cm)			
Scoring	2	1	0
Accuracy of needle entry	In the centre of the dot	Deviated from the centre	Outside the dot
Stability of the knot	Surgical triple knot	Smooth knot	Loose knot
Tissue integrity	Integrity	A few cracks	A complete fracture of the tissue
Tightness of the tissue	No gap between the two sides of the incision	A few gaps	Totally loose suture

### Statistical analysis

Six parameters were collected for analysis (suture time, thread length, accuracy of needle entry, stability of the knot, tissue integrity and tightness of the tissue), and the suture time was used for sample size calculation. Accoring to a similar research [10], a calculation with the width of the 95% confidence interval and an alpha level of 0.05 was performed, indicating that a minimum of 18 participants were required in each group to show a significant difference. The data are presented as the mean ± SD. The statistical analysis was performed with SPSS 18.0 statistical software. An independent t-test was used to analyse the differences between training groups, and the results was based on a two-sided test. *P* < 0.05 was considered to represent a statistically significant difference.

## Results

The forty residents were randomly divided into two groups, the control group and the experimental group, with twenty residents in each group. The control group included six males and fourteen females, while the experimental group included eight males and twelve females. The average age of the experimental group was 27.5 ± 0.89 years old, and the average age of the control group was 26.3 ± 0.95 years old. Whether they had video gaming experience was determined and recorded. Only one and two students in the control group and the experimental group, respectively, answered yes to this question (Table 2).

**Table 2:** Characteristics of the participants

	Average age (years old)	Sex (males/females)	Video gaming experience (the number of answer yes)
Experimental group	27.5 ± 0.89	8 / 12	2
Control group	26.3 ± 0.95	6 / 14	1

We also compared the basic surgical skills of the two groups. They both obtained low scores for the accuracy of needle entry, stability of the knot, tissue integrity, and tightness of the tissue. The suture also took quite a long time and a long thread for both groups. There was no statistically significant difference between the two groups before the training (*P* > 0.05) (Table 3).

**Table 3:** The data between the two groups before teaching

	Time (min)	Thread length (cm)	Overall score	Accuracy of needle entry	Stability of the knot	Tissue integrity	Tightness of the tissue
Experimental group	15.6 ± 1.70	23.1 ± 1.07	4.0 ± 0.73	0.75 ± 0.44	1.15 ± 0.37	0.90 ± 0.31	1.20 ± 0.41
Control group	14.7 ± 1.22	23.0 ± 1.10	3.7 ± 0.57	0.60 ± 0.50	1.10 ± 0.31	1.23 ± 0.32	1.78 ± 0.34
t	1.82	0.44	1.45	1.00	0.47	1.00	1.71
*P*	0.077	0.665	0.154	0.324	0.643	0.324	0.095

Data are mean ± SD

Overall score: The total score of the following 4 items

After the instructional programs, both groups improved for each item markedly. There were significant differences between the experimental group (7.9 ± 1.04 min) and the control group (11.6 ± 1.19 min) in terms of suture time (*P* = 0.001). As for accuracy of needle entry and exit, the experimental group acquired average score of 2.50 ± 0.51 and the control group got 1.85 ± 0.37 (*P* = 0.035). The experimental group got 2.43 ± 0.50 points and the control group got 1.41 ± 0.50 for tissue integrity (*P* = 0.030). However, groups’ performance on the knotting firmness (*P* = 0.241), the tightness of the suture tissue (*P* = 0.367), and the total thread length (*P* = 0.093) showed no significant difference (Table 4).

**Table 4:** The data between the two groups after teaching

	Time (min)	Thread length (cm)	Overall score	Accuracy of needle entry	Stability of the knot	Tissue integrity	Tightness of the tissue
Experimental group	7.9 ± 1.04	18.5 ± 1.58	7.5 ± 0.76	2.50 ± 0.51	1.31 ± 0.44	2.43 ± 0.50	1.37 ± 0.47
Control group	11.6 ± 1.19	19.4 ± 1.73	5.9 ± 0.55	1.85 ± 0.37	1.54 ± 0.60	1.41 ± 0.50	1.23 ± 0.37
t	10.62	1.73	7.38	4.61	1.19	6.29	1.13
*P*	*0.001	0.093	*0.027	*0.035	0.241	*0.030	0.367

Data are mean ± SD

Overall score: The total score of the following 4 items

*P < 0.05

## Discussion

Laparoscopic suturing is more arduous than open surgery suturing [11], and the suturing procedure for laparoscopy is challenging because laparoscopic surgery requires handling of specially designed tools and requires a small vision view to reduce the size of the wound, which is not the same as the conventional open method [12, 13]. It is not easy to control the needle and the distance and direction of the needle going in and out of the tissue. The surgeon’s hand movement is affected by the “pivot effect” under laparoscopy, which also increases the difficulty of laparoscopic operations [14]. In addition, finding the wound that needs stitching is done with the assistance of moving lens, which requires time and coordination; therefore, it is difficult to locate the wound and use the needle to suture a series of stitches quickly [15].

Laparoscopic suturing is regarded as the key skill in minimally invasive surgery. Growing attention has been paid to the laparoscopic skills of junior doctors. Different training programs with varying properties and modalities have been developed for laparoscopic suturing. Bansal et al. [16] oncluded that laparoscopic skill training under the microscope could improve suturing skill and help reduce the learning curve. Kockerling et al. [17] retrospectively analysed the concept of a simulation-based training -- participant evaluation course. “The curriculum for laparoscopy surgery” had three courses, each with a specific theme: a lecture about laparoscopic surgery, laparoscopic surgery technology training, and a biosimulator exercise. Several other studies have shown that students’ performance improves after training with laparoscopic simulators [18–21]. However, the optimal methods for teaching and training laparoscopic suturing techniques have not been defined.

In this study, on the basis of various scholars’ methods for improving laparoscopic suture teaching, we proposed the idea of multidirectional stitching technology in a laparoscopic suturing instructional program with a special circular suture training module. Different from traditional one-direction needle holding, varying needle-holding directions for the needle holder were used in the direction of needle pulling, and the needle-pulling direction was freely rotated in the suturing process to achieve free control of the needle pulling target, which improved the accuracy and freedom of suturing [22, 23].

How to assess students’ performance and scoring was one of the most prominent problems when we carried out the project. We chose the scoring system of the modified GESEA scoring method instead of the OSATS tool. The Objective Structured Assessment of Technical Skills (OSATS) tool is a classic tool to evaluate laparoscopic suture technology [24]. The main reason is that the OSATS tool focuses more on the evaluation of the traditional sewing method and the assessment of the needle holding position and direction. Our evaluation system is mainly designed for multidirectional stitching technology. It is different from previous systems in that it addresses the control of suture direction, the protection of suture tissue, and the evaluation of suture time (suture proficiency).

After the training course in this study, all the students in the course significantly improved their test scores compared with their pretraining scores. The data showed that there were significant differences between the groups in terms of the suturing time, the control of the suturing target, and the destruction of tissue. The suture time was significantly shortened, the needle delivery target was accurate, and the possibility of damaging suture tissue was reduced. Therefore, it indicated that undergoing laparoscopic simulation training could benefit junior surgeons before they involve into the clinical work.

We suppose that the multidirectional stitching method also has the potential to be applied in real clinical work, not only in laparoscopic teaching. It can reduce the chance of incorrect ligation and suturing and protect the integrity of tissue and organs. For example, it can decrease the possibility of tugging and tearing of blood vessels in difficult sutures, such as vascular sutures, and reduce the occurrence of intraoperative blood loss, postoperative thrombosis, and other complications.

Last, some limitations of our study should be mentioned. First, due to the fact that the number of participants enrolled in this study was small, we hope that the multidirectional stitching technology can be practiced and applied in more laparoscopic suturing teaching program. Second, we only compared this method with traditional teaching method. However, there are still other emerging researchs aimed to improve teaching efficiency and effects. We can further this study into comparison of different suturing approaches so as to find the most appropriate teaching mode.

## Conclusions

In summary, after we applied multidirectional stitching technology in the teaching of laparoscopic suturing, the participants showed an improvement in technical surgical skills. Therefore, multidirectional stitching technology might be a practicable, novel training method to acquire proficiency in manual laparoscopic skills in a training setting. This will enable trainees to pay more attention to the control of suture direction, the use of suture needles and the protection of the sutured tissue in the training course. We hope that multidirectional stitching technology can be used by more junior surgeons to achieve significant results.

## Data Availability

The datasets used and/or analyzed during the current study are available from the corresponding author on request.

## Abbreviations

CONSORT: Consohdated Standards of Reporting Trials
GESEA: Gynaecological Endoscopic Surgical Educaion and Assessment
OSATS: Objective Structured Assessment of Technical Skills

## References

[1] Peter SD, Ostlie DJ (2011). The necessity for prospective evidence for single-site umbilical laparoscopic surgery. Semin Pediatr Surg.

[2] Chen C, Zeng Q, Zhang N, Yu J, Yan D, Xu C (2019). Application of an extracorporeal-assisted Intracorporeal sliding knot-tying technique in minimally invasive surgery in children. J Laparoendosc Adv Surg Tech A.

[3] Jayadevan R, Stensland K, Small A, Hall S, Palese M. A protocol to recover needles lost during minimally invasive surgery. J Soc Laparoendosc Surg. 2014;18(4):e2014.00165. 10.4293/JSLS.2014.00165 PMC4254476 25489212

[4] Romero P, Nickel F, Mantel M, Frongia G, Rossler A, Kowalewski KF (2017). Intracorporal knot tying techniques - which is the right one?. J Pediatr Surg.

[5] Schmidt MW, Kowalewski KF, Trent SM, Benner L, Muller-Stich BP, Nickel F (2020). Self-directed training with e-learning using the first-person perspective for laparoscopic suturing and knot tying: a randomised controlled trial : learning from the surgeon's real perspective. Surg Endosc.

[6] Schmidt MW, Friedrich M, Kowalewski KF, De La Garza J, Bruckner T, Müller-Stich BP (2017). Learning from the surgeon's real perspective - First-person view versus laparoscopic view in e-learning for training of surgical skills? Study protocol for a randomized controlled trial. Int J Surg Protoc.

[7] Lin CC, Huang SC, Lin HH, Huang WJ, Chen WS, Yang SH (2018). Naked-eye box trainer and training box games have similar training effect as conventional video-based box trainer for novices: A randomized controlled trial. Am J Surg.

[8] Fjørtoft K, Konge L, Gögenur I, Thinggaard E (2019). The implementation gap in laparoscopic simulation training. Scand J Surg.

[9] Campo R, Wattiez A, Tanos V, Sardo ADS, Grimbizis G, Wallwiener D (2016). Gynaecological Endoscopic Surgical Education and Assessment. A diploma programme in gynaecological endoscopic surgery. Eur J Obstetrics Gynecol Reprod Biol.

[10] Abdelrahman M, Belramman A, Salem R, Patel B (2018). Acquiring basic and advanced laparoscopic skills in novices using two-dimensional (2D), three-dimensional (3D) and ultra-high definition (4K) vision systems: A randomized control study. Int J Surg (London, England).

[11] Agdi M, Tulandi T (2010). Minimally invasive approach for myomectomy. Semin Reprod Med.

[12] Sánchez-Margallo JA, Sánchez-Margallo FM, Oropesa I, Gómez EJ (2014). Systems and technologies for objective evaluation of technical skills in laparoscopic surgery. Minim Invasive Ther Allied Technol.

[13] Saito Y, Yamada S, Imura S, Morine Y, Ikemoto T, Iwahashi S (2018). A learning curve for laparoscopic liver resection: an effective training system and standardization of technique. Transl Gastroenterol Hepatol.

[14] Crothers IR, Gallagher AG, McClure N, James DT, McGuigan J (1999). Experienced laparoscopic surgeons are automated to the "fulcrum effect": an ergonomic demonstration. Endoscopy..

[15] Xiao J, Cui Z, Fu M, Kong X, Tang L, Wang Z (2016). An ex vivo liver training model continuously perfused to simulate bleeding for suture skills involved in laparoscopic liver resection: development and validity. Surg Endosc.

[16] Bansal VK, Tamang T, Misra MC, Prakash P, Rajan K, Bhattacharjee HK (2012). Laparoscopic suturing skills acquisition: a comparison between laparoscopy-exposed and laparoscopy-naive surgeons. J Soc Laparoendosc Surg.

[17] Köckerling F, Pass M, Brunner P, Hafermalz M, Grund S, Sauer J (2016). Simulation-Based Training - Evaluation of the Course Concept "Laparoscopic Surgery Curriculum" by the Participants. Front Surg.

[18] Hassan I, Koller M, Zielke A, Lehmann K, Rothmund M, Gerdes B (2006). Improvement of surgical skills after a three-day practical course for laparoscopic surgery. Swiss Med Wkly.

[19] Aggarwal R, Tully A, Grantcharov T, Larsen CR, Miskry T, Farthing A (2006). Virtual reality simulation training can improve technical skills during laparoscopic salpingectomy for ectopic pregnancy. BJOG.

[20] Calatayud D, Arora S, Aggarwal R, Kruglikova I, Schulze S, Funch-Jensen P (2010). Warm-up in a virtual reality environment improves performance in the operating room. Ann Surg.

[21] Maagaard M, Sorensen JL, Oestergaard J, Dalsgaard T, Grantcharov TP, Ottesen BS (2011). Retention of laparoscopic procedural skills acquired on a virtual-reality surgical trainer. Surg Endosc.

[22] Fairhurst K, Strickland A, Maddern G (2011). The LapSim virtual reality simulator: promising but not yet proven. Surg Endosc.

[23] Glasgow SC, Tiemann D, Frisella MM, Conroy G, Klingensmith ME (2006). Laparoscopy as an educational and recruiting tool. Am J Surg.

[24] Chang OH, King LP, Modest AM, Hur HC (2016). Developing an objective structured assessment of technical skills for laparoscopic suturing and Intracorporeal knot tying. J Surg Educ.

